# Neanderthal Extinction by Competitive Exclusion

**DOI:** 10.1371/journal.pone.0003972

**Published:** 2008-12-24

**Authors:** William E. Banks, Francesco d'Errico, A. Townsend Peterson, Masa Kageyama, Adriana Sima, Maria-Fernanda Sánchez-Goñi

**Affiliations:** 1 Institut de Préhistoire et de Géologie du Quaternaire, UMR 5199-PACEA, Université Bordeaux 1, CNRS, Talence, France; 2 Institute for Human Evolution, University of Witwatersrand, Johannesburg, South Africa; 3 Natural History Museum and Biodiversity Research Center, The University of Kansas, Lawrence, Kansas, United States of America; 4 Laboratoire des Sciences du Climat et de l'Environnement/IPSL, UMR 1572, CEA/CNRS/UVSQ, CE Saclay, L'Orme des Merisiers, Gif-sur-Yvette, France; 5 EPHE, UMR5805-EPOC, Université Bordeaux 1, CNRS, Talence, France; University of Utah, United States of America

## Abstract

**Background:**

Despite a long history of investigation, considerable debate revolves around whether Neanderthals became extinct because of climate change or competition with anatomically modern humans (AMH).

**Methodology/Principal Findings:**

We apply a new methodology integrating archaeological and chronological data with high-resolution paleoclimatic simulations to define eco-cultural niches associated with Neanderthal and AMH adaptive systems during alternating cold and mild phases of Marine Isotope Stage 3. Our results indicate that Neanderthals and AMH exploited similar niches, and may have continued to do so in the absence of contact.

**Conclusions/Significance:**

The southerly contraction of Neanderthal range in southwestern Europe during Greenland Interstadial 8 was not due to climate change or a change in adaptation, but rather concurrent AMH geographic expansion appears to have produced competition that led to Neanderthal extinction.

## Introduction

Climate changes unquestionably influenced Paleolithic hunter-gatherer adaptations, and particular attention has been paid to possible climatic influences on Neanderthal extinction and colonization of Europe by anatomically modern humans (AMH) [1]–[4]. Reasons behind Neanderthal extinction, however, are still debated intensively. Two competing hypotheses contend either that Neanderthals were unable to adapt to climatic changes towards the end of Marine Isotope Stage 3 (MIS3) or that competition with AMH was the driving factor in their extinction.

MIS3 (60–30 kyr cal BP), marked by many of the largest and quickest temperature excursions of the last glacial period [5], was characterized by an ice sheet of intermediate size and intermediate atmospheric CO_2_ concentrations. MIS3 was punctuated by periods, called Heinrich events [6], during which massive discharges of icebergs into the Northern Atlantic Ocean resulted in near shut-down of the Atlantic Meridional Overturning Circulation [7]. Associated decreases in mid-latitude North Atlantic sea surface temperatures had marked rapid impacts on continental climate and vegetation. Greenland Interstadials (GI; mild phases) were characterized in Western Europe by open forest landscapes, while herbaceous-dominated landscapes existed during Greenland Stadials (cold phases) [8]. The environmental conditions associated with such phases, and the rapid and marked transitions between them, likely affected the distributions and adaptations of human populations.

Considerable discussion has surrounded the disappearance of Neanderthals and the spread of AMH, with debate focused on a number of specific issues: (a) relationships between particular stone tool technologies, or archaeologically-defined cultures (termed technocomplexes), and the human populations who made them (i.e., Neanderthals or AMH); (b) possible cultural interactions between these two human populations; (c) mechanisms behind Neanderthal extinction; and (d) timing of this population event.

With respect to the authorship of archaeological assemblages dated to ∼43–35k calibrated (calendar) years ago (kyr cal BP), consensus exists that, in Europe, Mousterian technocomplexes were solely manufactured by Neanderthals [cf. 9], [10]. Most agree that the Châtelperronian, the only ‘transitional technocomplex’ associated with diagnostic human remains was also made by Neanderthals [11]–[13] ―we assume this to be the case for the Bohunician [14] ―, and that the typical Aurignacian technocomplex should be attributed to AMH [cf. 2], [15].

Intense debate has focused on possible cultural interactions between Neanderthal and AMH populations. Reappraisals of key sites have challenged the existence of a diagnostic Aurignacian older than ∼41 kyr cal BP in Western Europe [16], [17] and have shown that the Châtelperronian, previously interpreted as representing acculturation of Neanderthals by AMH immigrants, is almost certainly older than the first Aurignacian [18], [19]. This assertion is consistent with the fact that the most recent reliably dated Mousterian sites in France are not younger than ∼40.5 kyr cal BP [20] and that the Châtelperronian does not post-date ∼40.5–39 kyr cal BP [19]. Although this timeline is now supported widely [21], [22], some still consider the evidence ambiguous [23], [24], and others support the idea of an early colonization of Europe by AMH at ∼43 kyr cal BP, with subsequent acculturation of late Neanderthal populations prior to their extinction [4], [9], [25]–[28]. Some have also suggested the possibility of Neanderthal biological input, albeit undetected by genetic studies [29]–[32], to the first wave of AMH colonizers [2], [33], [34].

Considerable research links Neanderthal decline and extinction with MIS3 environmental variability, in particular regarding population dynamics during specific Dansgaard-Oeschger (D-O) climatic phases. Consensus exists that Neanderthal populations persisted in southern Europe, particularly in southern Iberia, well after they had disappeared from northern latitudes, and that environmental conditions briefly created a geographic barrier between them and AMH called the Ebro Frontier [35].

Diverse methodological approaches have been used to integrate paleoclimatic, chronological, and archaeological datasets [36], [37] in efforts to understand human population dynamics during this period, and discussions have also focused on limitations of radiocarbon dating [24], [38]–[41]. By correlating palynological data from deep sea cores with archaeological data, it has been proposed that AMH were present in Western Europe and northern Iberia just prior to Heinrich event 4, that conditions during Heinrich event 4 delayed their colonization of southern Iberia, and that subsequent competition with AMH drove Neanderthal extinction after this climatic episode [20]. A very late (∼32 kyr cal BP) survival of Neanderthals in southern refugia, based on dates from Gorham's Cave, Gibraltar, has been proposed [42], and an even later disappearance (22.5–25.5 kyr cal BP) has been suggested recently [43]. This last proposal contends that D-O variability did not have a significant impact on this region, but rather that the long-term trend towards less favorable environmental conditions stressed Neanderthals to extinction, with little or no impact of competition with AMH. Such an idea, however, is contradicted by high-resolution climatic and vegetation simulations for Heinrich event 4 [44], which suggest development of semi-desert conditions in central and southern Iberia that impacted Neanderthal populations and delayed AMH settlement and consequent competition.

Creating a consensual chronological framework for the Middle-to-Upper Paleolithic transition is complicated by limitations of radiocarbon dating, uncertainties in radiocarbon comparison curves, and fluctuations in ^14^C levels [38], [39]. Recent dating methods have shown that ages from many previously dated samples underestimate true ages [40], [41], and disagreements exist on cultural attributions assigned to archaeological levels at key sites. These discussions are complicated by the fact that correlating cultural and climatic events during MIS3 is difficult because the former are in radiocarbon years while some of the latter are in calendar years and often span relatively short periods of time (∼1500 yr). Only recently have systematic efforts been made to overcome these limitations, either by correlating archaeological data directly with long, radiocarbon-dated climatic sequences [20], [36] or by using comparison curves to ‘calibrate’ radiocarbon ages before correlating them with paleoclimatic sequences [26], [45].

Here, we apply a new method that incorporates a variety of diverse data sets to reflect on this important population event to evaluate the climate versus competition hypotheses for Neanderthal extinction. Recent advances in biodiversity studies [46] have developed tools for estimating ecological niches of species and predicting responses to environmental changes. These tools were originally developed to estimate ecological niches of species and predict responses to environmental changes. It has been recently shown that they have considerable potential for reconstructing eco-cultural niches of past human populations [47], defined as the potential range of environmental conditions within which a human adaptive system can exist without having to undergo significant change. Our assumption is that human adaptive systems, defined here as the range of technological and settlement systems shared and transmitted by a culturally cohesive population within a specific paleoenvironmental framework, can be considered to operate as a ‘species’ with respect to their interaction with the environment. This does not imply, however, that human adaptive systems necessarily remained stable over time, as might be the case with animal species occupying narrow and stable niches. Humans can change their adaptive systems rapidly through technical and social innovations in response to environmental change. We know, however, that this was not the case during the late Middle and Upper Paleolithic, periods during which specific human adaptive systems spanned a number of climatic events. Thus, the method described in this study is particularly relevant for addressing issues of human adaptive system stability and eco-cultural niche stability. Another advantage of this methodology is that it can help identify mechanisms (i.e. niche conservatism, niche contraction, etc.) behind changes occurring across time and space in the relationship between adaptive systems and environments by projecting a reconstructed human eco-cultural niche into a different paleoenvironmental framework.

We focus on the three climatic phases during which the bulk of AMH colonization of Europe and Neanderthal contraction (if not extinction) occurred: Greenland Interstadials 9–11 (pre-H4; 43.3–40.2 kyr cal BP, see [48]), Heinrich event 4 (H4; 40.2–38.6 kyr cal BP), and Greenland Interstadial 8 (GI8; 38.6–36.5 kyr cal BP). GI9–11 were three short-term mild events separated by two brief periods of cooling. They were marked by relatively wet conditions in Atlantic regions of Europe and comparatively drier conditions in western Mediterranean regions. H4 was marked in the western Mediterranean by extremely cold and dry conditions resulting in semi-desert vegetation, but was not so arid farther north with a consequent expansion of grasslands. GI8 was a relatively long phase with mild, moist conditions along Atlantic margins, which led to a weak development of deciduous forests. In western Mediterranean regions, warm, dry summers and moist winters created an open Mediterranean forest [8].

Here, we apply the approach termed eco-cultural niche modeling (ECNM; see *Materials and Methods* below) [49], to late Neanderthal and early AMH adaptive systems to define and characterize eco-cultural niches associated with these populations for each relevant climatic event, evaluate whether these niches changed during the Middle-to-Upper Paleolithic transition, and evaluate whether climate change or competition with AMH caused Neanderthal extinction.

## Results

**Figure 1:**
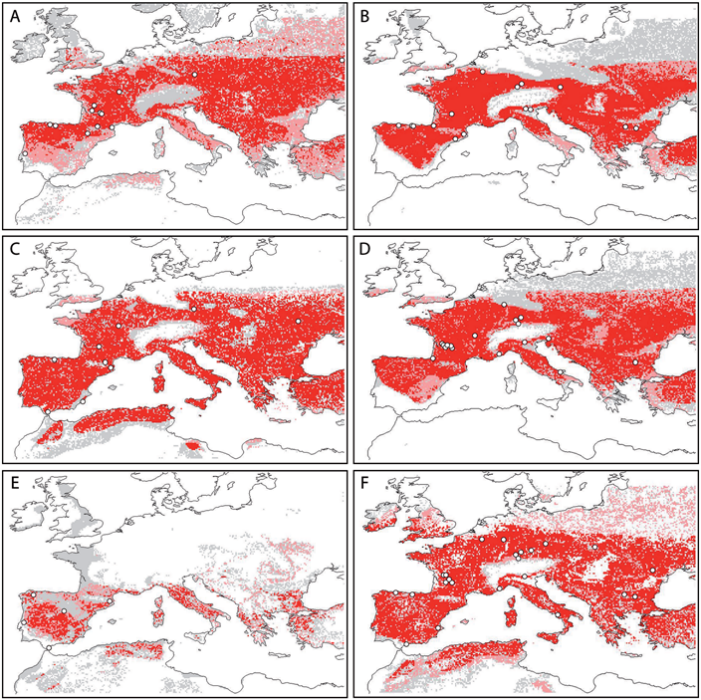
Maps of geographic projections of conditions identified as suitable by eco-cultural niche models for Neanderthals (A – pre-H4, C – H4, E – GI8) and AMH (B – pre-H4, D – H4, F – GI8). Grid squares with 1–5 of 10 models predicting presence of suitable conditions are indicated in grey, grid squares with 6–9 models in agreement are depicted in pink, and squares with all 10 models in agreement are indicated in red. Archaeological site locations are indicated with circles.

The ECNM for the pre-H4 Neanderthal adaptive system (Figure 1A) shows a potential distribution across ∼40°–∼50°N latitude, excepting the Alps and the Po and terminal Danube River plains. Suitability in Mediterranean regions is generally estimated as lower. Climatically, the predicted niche occupies a mean annual temperature range of −1°–+12°C and precipitation of <1095 mm/yr. The pre-H4 niche for AMH (Figure 1B) does not extend as far north as that of Neanderthals (Figure 1A), includes a tongue of potential distributional area extending into southeastern Iberia, and lacks suitable areas in southwestern Iberia. The pre-H4 AMH niche occupies a slightly narrower temperature range, but with precipitation values virtually identical to those of Neanderthals. The H4 Neanderthal potential distribution (Figure 1C) is reconstructed as occupying the entire Iberian, Italian, and Balkan peninsulas, with sharply defined northern limits, covering mean annual temperatures of 0–10°C and precipitation <730 mm/yr. The H4 AMH distribution (Figure 1D) again did not include southwestern Iberia, but has northern range limits and environmental ranges similar to those of the H4 Neanderthal adaptive system. The Neanderthal GI8 model, however, indicates a dramatically reduced potential distributional area, restricted to Mediterranean regions (Figure 1E). This niche occupies a mean annual temperature of 6–14°C with precipitation of <730 mm/yr. In contrast, the AMH GI8 model (Figure 1F) covers most of central and southern Europe, including a broader temperature (0–15°C) and precipitation (<1095 mm/yr) range than the contemporaneous Neanderthal niche. Principal component analyses performed on all the environmental variables associated with each of the six ECNMs all indicated that temperature variables were the most important in defining ranges of both adaptive systems. Almost all models showed significant predictive ability based on jackknife manipulations within time periods (all *P*<0.05, except for H4 and GI8 Neanderthals, the periods with smallest sample size and most restricted distributions).

**Figure 2:**
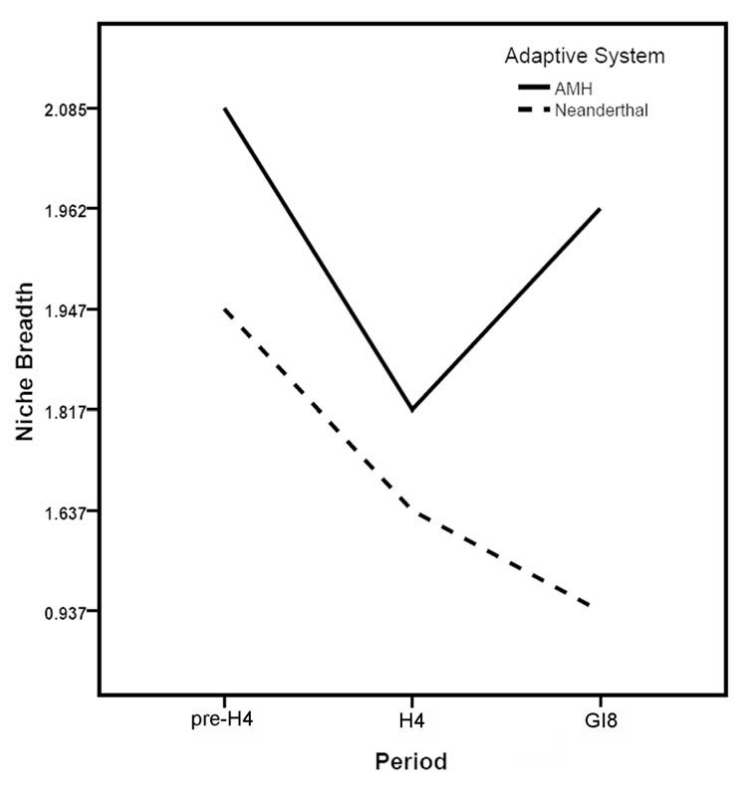
Summary of niche breadth measures for Neanderthal and AMH adaptive systems during each of the three climatic phases examined.

Neanderthal ECNM niche projections were able to predict the distribution of this adaptive system from pre-H4 to H4 and H4 to GI8 (Table 1) better than random expectations (*P*<0.05). This result suggests that Neanderthals exploited the same eco-cultural niche across the three climatic phases, or at least that the niche had not shifted dramatically. For AMH as well, inter-period projections were statistically significantly interpredictive (Table 1). Niche breadth is similar between the two adaptive systems for pre-H4 and H4; however, during GI8, AMH niche breadth increases markedly but Neanderthal niche breadth decreases considerably (Figure 2).

**Table 1:** Results of tests of predictivity among three climatic phases for Neanderthal and AMH eco-cultural niche model projections.

Comparison	All models predict			Most models predict			Any model predicts		
	Proportional Area	Success	*P*	Proportional Area	Success	*P*	Proportional Area	Success	*P*
Neanderthal pre-H4 predicts H4	0.2303	1/9	0.6499	0.3798	4/9	0.2259	0.584	8/9	0.0079
Neanderthal H4 predicts GI8	0.4599	3/5	0.1415	0.5651	4/5	0.0576	0.6452	4/5	0.1118
AMH pre-H4 predicts H4	0.2498	11/17	0.0001	0.3463	12/17	0.0005	0.432	13/17	0.0011
AMH H4 predicts GI8	0.3616	15/24	0.0023	0.4637	20/24	0.00003	0.6003	21/24	0.0007

## Discussion

**Figure 3:**
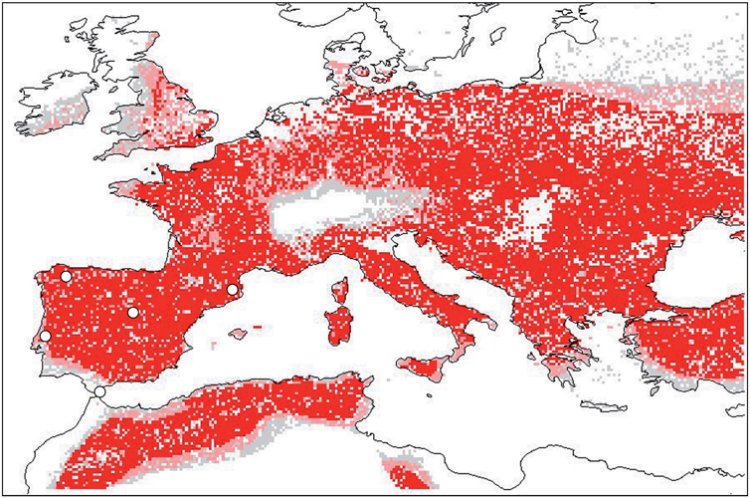
Projection of the H4 Neanderthal model onto GI8 climatic conditions. Grid squares with 1–5 of 10 models predicting presence of suitable conditions are indicated in grey, grid squares with 6–9 models in agreement are depicted in pink, and squares with all 10 models in agreement are indicated in red. Neanderthal sites dated to GI8 are indicated with circles.

Our results highlight a reduction of potential Neanderthal range from pre-H4 through GI8, in terms of both ecology and geography. Two contrasting explanations were discussed above: (1) a contracting geographic footprint of the same niche in response to changing climate, versus (2) competition with expanding AMH populations. The first hypothesis implies that Neanderthals exploited the same ecological niche throughout the three climatic phases but had reduced geographic potential as the spatial manifestation of that niche contracted due to climate change. This scenario, however, can be rejected because the H4 to GI8 projection shows that the climatic shift to warmer and wetter conditions during GI8 anticipated a broader distributional area (Figure 3). This result indicates that only a small part of Neanderthal potential range was exploited during GI8, and that this reduced range was not a result of a contracting suitable climatic footprint, contradicting recent proposals that Early Upper Paleolithic populations reduced their niche due to environmental stress [50].

Our results indicate instead that competition with AMH represents a more cogent explanation for the situation. Predicted niches and potential geographic distributions for Neanderthal and AMH adaptive systems overlap broadly during pre-H4 and H4, except that southern Iberia was not within the distributional potential of AMH, lending support to the notion that the Ebro Frontier resulted from ecological causes. During GI8, however, AMH niche breadth and potential distribution broadened, permitting AMH exploitation of the last Neanderthal refugium. The AMH expansion and Neanderthal contraction of niche characteristics were concurrent, and we suspect causally related. It follows that there was certainly contact between the two populations, which may have permitted both cultural and genetic exchanges. Our findings clearly contradict the idea that Neanderthal demise was mostly or uniquely due to climate change [51] and looks towards AMH expansion as the principal factor. Hence, we contend that AMH expansion resulted in competition with which the Neanderthal adaptive system was unable to cope.

## Materials and Methods

To reconstruct eco-cultural niches, we used the Genetic Algorithm for Rule-Set Prediction (GARP) [52], which has been applied to topics as diverse as habitat conservation, the effects of climate change on species' distributions, the geographic potential of species' invasions, and the geography of emerging disease transmission risk [53]–[57]. It is available for free download at http://www.nhm.ku.edu/desktopgarp/. For data inputs, GARP requires the geographic coordinates where the target species has been observed and raster GIS data layers summarizing landscape and climatic dimensions potentially relevant to shaping the distribution of the species.

In this case, the ‘species’ is a technological adaptive system. Here, the occurrence data are the geographic coordinates of radiometrically dated and culturally attributed archaeological sites. These archaeological data were obtained from a database [58] containing the geographic coordinates, recorded stratigraphic levels, and cultural affiliations associated with ∼6000 radiometric ages from ∼1300 archaeological sites across Europe. The late Middle Paleolithic and early Upper Paleolithic technocomplexes date to the temporal limits of radiocarbon methods, making their ^14^C determinations particularly sensitive to contamination by more recent carbon sources, resulting in frequent underestimation of true ages of samples [16], [20], [40], [59], [60]. For this reason, we restricted the site samples used to create our pre-H4, H4, and GI8 ECNMs to sites dated by accelerator mass spectrometry (AMS) and containing diagnostic archaeological assemblages from stratified contexts, with a single exception (Table S1). Some AMS ages have relatively large associated errors such that it is difficult, if not impossible, to be sure that they date an occupation during a specific climatic event. Such ages were eliminated from consideration for this study. Also, it has been shown that a number of ages come from archaeological levels that have likely been disturbed by post-depositional site formation processes and it is unclear if the dated material was originally associated with the archaeological level from which it was recovered [see 45]. In these instances as well, the AMS ages in question were not used in this analysis. These quality-control steps minimize the possibility of incorporating sites for which radiometric determinations are minimum ages, and increase the likelihood that dates reflect a human presence during a specific climatic event. We employed CalPal [61] (using the recent Greenland-Hulu comparison curve [62]) to calibrate the age determinations and assign them to specific climatic phases.

It has been proposed [24] that any use of radiocarbon ages for this time period should be considered provisional see also [63]. We do not think, however, that a careful and consistent selection of dates will necessarily result in erroneous or misleading conclusions. Additionally, our method of testing model predictivity (see below) allows us to identify sites inconsistent with the remainder of the sample attributed to a particular climatic phase. In short, we need to test the pertinence of new methodological approaches on the available archaeological and chronological datasets so that heuristic tools will be in place as new data emerge.

The environmental data sets consisted of topographic/landscape attributes (assumed to have remained constant) and high-resolution climatic simulations for the three climatic phases considered here. Landscape variables included slope, aspect, and compound topographic index (a measure of tendency to pool water) from the Hydro-1K dataset (U.S. Geological Survey's Center for Earth Resources Observation and Science - http://edc.usgs.gov/products/elevation/gtopo30/hydro/index.html).

The climatic simulations were created using the LMDZ3.3 Atmospheric General Circulation Model [64], in a high-resolution version (144 cells in longitude×108 in latitude), with further refinement over Europe (final resolution ∼50 km) obtained by use of a stretched grid. Three simulations were performed with boundary conditions representing the three typical climatic situations of interest here: pre-H4 (baseline), interstadial, and Heinrich event, with mid-size ice-sheets compared to the full Last Glacial Maximum. Common to all simulations are the ice-sheets imposed as boundary conditions for which we used the Peltier [65] ICE-4G reconstructions for 14 kyr cal BP, a time at which sea-level was similar to that of Marine Isotope Stage 3 for which no global reconstructions exist. Orbital parameters and greenhouse gas concentrations were set to their 40 kyr cal BP values [44].

The only difference between the three simulations concerned sea surface temperatures (SSTs) and sea-ice extent in the North Atlantic. For the baseline configuration, we used the GLAMAP reconstruction [66]. For the Heinrich event configuration, we subtracted from the reference SSTs an anomaly of 2°C in the mid-latitude North Atlantic and Mediterranean Sea. The interstadial configuration added an anomaly of 2°C to the reference SSTs in the mid-latitude North Atlantic and Mediterranean Sea. For both states, sea-ice cover is imposed if SSTs are lower than −1.8°C. The model was then run with these boundary conditions for 21 years, the last 20 of which were used to compute atmospheric circulation and surface climate in balance with our defined boundary conditions. European climate proves quite sensitive to these changes in boundary conditions: continental temperatures and precipitation decrease from the interstadial to the stadial and finally the Heinrich event simulations, in a fashion similar to results described elsewhere [44]. From these climate simulations, temperature (the coldest and the warmest months as well as mean annual temperature) and precipitation values were extracted for use in GARP. The baseline simulation was used as a proxy for conditions during the period covering Greenland Interstadials 9–11 (pre-H4). The Heinrich event simulation is used to represent conditions during Heinrich event 4 (H4), and the interstadial simulation represents Greenland Interstadial 8 (GI8).

This experiment set-up is designed to be as realistic as possible for MIS3, given the available global data sets needed to perform atmosphere-only experiments. We used more recent SST/sea-ice reconstructions for our baseline experiment compared to previous simulations for the same climatic events [44]. In particular, these reconstructions are warmer over the North Atlantic than the CLIMAP [67] reconstruction and thus more relevant for the MIS3 baseline simulation. Therefore, the climate simulations used in the present study are unique for several reasons: they use updated SST reconstructions, mid-size ice-sheets, greenhouse gas levels, and orbital parameters appropriate for the periods that bracket Heinrich event 4. The resulting climate is obviously dependent on the hypotheses built up in the boundary conditions we used, and on the climate model itself, but we do not know of any equivalent experiments, with an equivalent model, that have high resolution over Europe.

In GARP, occurrence data are resampled randomly by the algorithm to create training and test data sets. An iterative process of rule generation and improvement then follows, in which an inferential tool is chosen from a suite of possibilities (e.g., logistic regression, bioclimatic rules) and applied to the training data to develop specific rules [52]. These rules are then “evolved” to maximize predictivity by using a number of methods (e.g. crossing over among rules), mimicking chromosomal evolution. Predictive accuracy is evaluated based on the presence data and a set of points sampled randomly from regions where the species has not been detected. The resulting rule-set defines the distribution of the subject in ecological space (i.e., an ecological niche) [68] and can be projected onto the landscape to predict a potential geographic distribution [69].

We used the following specifications in GARP. Given the random-walk nature of the method, we ran 1000 replicate runs, with a convergence limit of 0.01. Given the small sample sizes (*N*), we used *N* − 2 occurrence points to develop models in each analysis, reserving one point for model selection and one for evaluating model predictive ability. We followed a modification of a protocol for selecting among resulting models [70], with omission error (i.e., failure to predict a known presence) measured based on the single reserved model-selection point, and models retained only when they were able to predict that single point (i.e., hard omission threshold of 0%). Commission error, conversely, is a measure of areas of absence that are incorrectly predicted present; we followed recommendations of removing from consideration those 50% of models that show extreme values of proportional area predicted present. The resulting 10 final ‘best subset’ models were then summed pixel by pixel to produce a best estimate of an adaptive system's potential geographic distribution. This conservative approach is ideal when working with small sample sizes, and helps to maximize the robustness of the prediction.

Predictive models such as ECNMs are just that—predictions that must be tested for predictive accuracy before they can be interpreted. Given low occurrence data samples, we tested model predictions using the jackknife manipulation proposed by Pearson et al. [71], the only robust test for evaluating models based on small samples: *N*−1 points are used to develop *N* jackknifed models. The success of each replicate model in predicting the single omitted point, relative to the proportional area predicted present, is then calculated using an extension to the cumulative binomial probability distribution.

To determine if the Neanderthal and AMH adaptive systems exploited different environmental regimes, their predicted eco-cultural niches, plotted in ecological space against available climatic data, were reviewed for each climatic phase. To determine which environmental variables most influenced the reconstructed niches, principal component analyses (PCA) were performed on these same data (climatic and geographic variables) for each period using *SPSS* 16.0.

We employed the GARP capability to project the ecological niche predicted for a climatic phase onto the environmental conditions of a subsequent period to evaluate if an adaptive system exploited the same ecological niche across different climatic phases (i.e., niche conservation). The resulting projection is compared to the locations of known occurrences for the latter period to see whether or not the model successfully predicts their spatial distribution. The degree of predictivity (i.e., niche stability) was evaluated statistically by determining the proportional area predicted present by the projected model at each predictive threshold (i.e., 10 out 10 best subset models in agreement, 9 out of 10 in agreement, etc.) along with the number of occurrence points correctly predicted at each threshold. A cumulative binomial statistic is applied to these values to determine whether the coincidence between projected predictions and independent test points is significantly better than random expectations (Table 1). In other words, this approach evaluates whether the two distributions are more similar to one another than one would expect by chance.

To further examine variability within and between eco-cultural niches, we calculated a measure of niche breadth as the sum of the variances along independent factor axes [72], [73]. First, predictions for each adaptive system and each climatic phase were projected with GARP onto the climatic variables associated with GI8. We performed a PCA on the GI8 climatic variables, and retained sufficient factors to explain 99% of the overall variance (*N* = 3). Then, the variance of the factor loadings associated with areas predicted present by all 10 best subset models was calculated along each principal component and then summed across them. This sum is a robust measure of niche breadth, defined as the diversity of abiotic conditions under which a species can maintain a population [72], [74].

## Supporting Information

Table S1 Archaeological sites with radiometrically dated components attributed to Neanderthals (Mousterian, Châtelperronian, Bohunician) or AMH (Aurignacian) for the pre-H4, H4, and GI8 climatic phases.
(0.18 MB DOC)
