# Supplementary material for: Neanderthal Extinction by Competitive Exclusion
Source: PLoS One. 2008 Dec 24;3(12):e3972. doi: 10.1371/journal.pone.0003972 (PMC2600607; doi:10.1371/journal.pone.0003972)
Supplement: Table S1 — Archaeological sites with radiometrically dated components attributed to Neanderthals (Mousterian, Châtelperronian, Bohunician) or AMH (Aurignacian) for the pre-H4, H4, and GI8 climatic phases. (0.18 MB DOC) [file pone.0003972.s001.doc]

Table S1: Archaeological sites with radiometrically dated components attributed to Neanderthals (Mousterian, Châtelperronian, Bohunician) or AMH (Aurignacian) for the pre-H4, H4, and GI8 climatic phases.

| Site | Culture | Long. | Lat. | Country | Code | | Date | SD | cal BP | Type |
| --- | --- | --- | --- | --- | --- | --- | --- | --- | --- | --- |
| Greenland Interstadials 9–11 (pre-H4) | | | | | | | | | | |
| Arrillor | Mousterian | 0.94 | 43.01 | Spain | OxA-6106 | | 37000 | 1000 | 41900 | AMS |
| Combe Sauniere | Chatelperronian | 0.16 | 45.14 | France | no code | | 38100 | 1000 | 42490 | AMS |
| Enl Sidron | Mousterian | -5.3 | 43.38 | Spain | Beta-192066 | | 37300 | 830 | 42040 | AMS |
| Esquilleu | Mousterian | -4.6 | 43.2 | Spain | AA-37882 | | 36500 | 830 | 41140 | AMS |
| Grotte des Fees | Chatelperronian | 3.63 | 46.39 | France | OxA-14320 | | 39240 | 380 | 43120 | AMS |
| Grotte du Renne | Chatelperronian | 3.75 | 47.62 | France | OxA-8451 | | 38300 | 1300 | 42650 | AMS |
| Grotte XVI | Chatelperronian | 1.2 | 44.8 | France | AA-2997 | | 38100 | 1670 | 42430 | AMS |
| La Quina | Chatelperronian | 0.45 | 45.82 | France | OxA-10261 | | 35950 | 450 | 40800 | AMS |
| Las Fuentes | Mousterian | -0.41 | 42.13 | Spain | OxA-8524 | | 36050 | 550 | 40840 | AMS |
| Mezmaiskaya | Mousterian | 32.99 | 51.72 | Russia | Beta-53897 | | 36280 | 540 | 41440 | AMS |
| Oliveira | Mousterian | -8.62 | 39.5 | Portugal | GrA-9760 | | 38390 | 480 | 42610 | AMS |
| Roc de Combe | Chatelperronian | 1.35 | 44.75 | France | OxA-1443 | | 38000 | 2000 | 41950 | AMS |
| Stranska Skala | Bohunician | 13.68 | 49.82 | Czech Rep. | AA-41478 | | 36350 | 990 | 40830 | AMS |
| Arbreda | Aurignacian | 2.75 | 42.17 | Spain | OxA-3729 | | 37340 | 1000 | 42030 | AMS |
| Bacho Kiro | Aurignacian | 25.42 | 42.93 | Bulgaria | OxA-3183 | | 37650 | 1450 | 42080 | AMS |
| Caminade | Aurignacian | 1.25 | 44.87 | France | GifA-97185 | | 37200 | 1500 | 41320 | AMS |
| Castillo | Aurignacian | -3.97 | 43.29 | Spain | AA-2407 | | 37700 | 1800 | 41700 | AMS |
| Fumane | Aurignacian | 10.88 | 45.55 | Italy | Utc-2048 | | 36500 | 600 | 41600 | AMS |
| Geissenklosterle | Aurignacian | 9.78 | 48.4 | Germany | KIA-16032 | | 36560 | 410 | 41680 | AMS |
| Hohlenstein Stadel | Aurignacian | 10.38 | 48.77 | Germany | KIA-8950 | | 36910 | 490 | 41860 | AMS |
| Isturitz | Aurignacian | -1.2 | 43.37 | France | GifA-98232 | | 36510 | 610 | 41600 | AMS |
| La Vina | Aurignacian | -5.82 | 43.31 | Spain | Ly-6390 | | 36500 | 750 | 41560 | ^14^C |
| Morin | Aurignacian | -3.82 | 43.36 | Spain | GifA-96263 | | 36590 | 770 | 41560 | AMS |
| Paina | Aurignacian | 11.52 | 45.43 | Italy | UtC-2042 | | 37900 | 800 | 42370 | AMS |
| Romani | Aurignacian | 1.67 | 41.54 | Spain | AA-6608 | | 36740 | 920 | 41540 | AMS |
| Temnata | Aurignacian | 24.05 | 43.17 | Bulgaria | no code | | 36900 | 1300 | 41140 | AMS |
| Trou Al Wesse | Aurignacian | 5.25 | 50.47 | Belgium | OxA-7634 | | 36500 | 1100 | 40900 | AMS |
| Willendorf | Aurignacian | 15.4 | 48.32 | Austria | GrA-896 | | 37930 | 750 | 42380 | AMS |
| Heinrich event 4 (H4) | | | | | | | | | | |
| Arbreda | Mousterian | 2.75 | 42.17 | Spain | AA-3777 | | 34100 | 700 | 39160 | AMS |
| Belvis | Chatelperronian | 2.08 | 42.85 | France | AA-7390 | | 35425 | 1140 | 40110 | AMS |
| Buzdujeni | Mousterian | 27.3 | 48.17 | Moldova | no code | | 35400 | 1400 | 39910 | AMS |
| Cabezo Gordo | Mousterian | -0.95 | 37.73 | Spain | OxA-10666 | | 34450 | 600 | 39690 | AMS |
| Esquilleu | Mousterian | -4.6 | 43.2 | Spain | AA-37883 | | 34380 | 670 | 39580 | AMS |
| Gorham’s Cave | Mousterian | -5.35 | 36.21 | Spain | OxA-10295 | | 34600 | 900 | 39590 | AMS |
| Grotte du Renne | Chatelperronian | 3.75 | 47.62 | France | OxA-8452 | | 34450 | 750 | 39580 | AMS |
| Grotte XVI | Chatelperronian | 1.2 | 44.8 | France | GifA-95581 | | 35000 | 1200 | 39700 | AMS |
| Stranska Skala | Bohunician | 13.68 | 49.82 | Czech Rep. | AA-41477 | | 34530 | 770 | 39640 | AMS |
| Bacho Kiro | Aurignacian | 25.42 | 42.93 | Bulgaria | OxA-3212 | | 34800 | 1150 | 39530 | AMS |
| Caminade | Aurignacian | 1.25 | 44.87 | France | GifA-97186 | | 35400 | 1100 | 40110 | AMS |
| Castanet | Aurignacian | 1.18 | 45.03 | France | GifA-97312 | | 34800 | 1100 | 39590 | AMS |
| Combe Sauniere | Aurignacian | 0.16 | 45.14 | France | OxA-6507 | | 34000 | 850 | 38850 | AMS |
| Divje Babe | Aurignacian | 14.06 | 46.00 | Slovenia | RIDDL-734 | | 35300 | 700 | 40210 | AMS |
| Flageolet | Aurignacian | 0.58 | 44.82 | France | GifA-95559 | | 34300 | 1100 | 38980 | AMS |
| Fumane | Aurignacian | 10.88 | 45.55 | Italy | LTL-375A | | 34312 | 347 | 39700 | AMS |
| Geissenklosterle | Aurignacian | 9.78 | 48.4 | Germany | KIA-8959 | | 34220 | 310 | 39660 | AMS |
| Hohle Fels | Aurignacian | 9.73 | 48.37 | Germany | KIA-18880 | | 34190 | 340 | 39630 | AMS |
| Hohlenstein Stadel | Aurignacian | 10.38 | 48.77 | Germany | KIA-8949 | | 33920 | 310 | 39420 | AMS |
| Isturitz | Aurignacian | -1.2 | 43.37 | France | GifA-98233 | | 34630 | 560 | 39830 | AMS |
| Mochi | Aurignacian | 7.53 | 43.78 | Italy | OxA-3592 | | 34870 | 800 | 39890 | AMS |
| Paglicci | Aurignacian | 15.58 | 41.67 | Italy | Utrecht | | 34300 | 800 | 39180 | AMS |
| Pataud | Aurignacian | 0.38 | 44.90 | France | GrN-4507 | | 34250 | 675 | 39450 | AMS |
| Solutre | Aurignacian | 4.31 | 46.38 | France | SRLA-058 | | 33970 | 360 | 39440 | AMS |
| Vogelherd | Aurignacian | 10.07 | 47.95 | Germany | PL-1342A | | 34100 | 1100 | 38810 | AMS |
| Wildscheuer | Aurignacian | 8.17 | 50.4 | Germany | OxA-7394 | | 34200 | 900 | 39040 | AMS |
| Greenland Interstadial 8 (GI8) | | | | | | | | | | |
| A Valina | Chatelperronian | -7.34 | 43.05 | Spain | GrA-3014 | 31600 | | 250 | 35460 | AMS |
| Almonda | Mousterian | -8.62 | 39.5 | Portugal | OxA-8671 | 32740 | | 420 | 37100 | AMS |
| Ermitons | Mousterian | 2.65 | 42.23 | Spain | OxA-3725 | 33190 | | 660 | 37700 | AMS |
| Gorham’s Cave | Mousterian | -5.35 | 36.13 | Spain | OxA-7857 | 32280 | | 420 | 36720 | AMS |
| Jarama | Mousterian | -3.33 | 40.92 | Spain | Beta-56639 | 32600 | | 1800 | 37560 | AMS |
| Oliveira | Mousterian | -8.62 | 39.50 | Portugal | GrA-10200 | 32740 | | 420 | 37100 | AMS |
| Bacho Kiro | Aurignacian | 25.42 | 42.93 | Bulgaria | OxA-3181 | 32200 | | 780 | 36660 | AMS |
| Bajondillo | Aurignacian | -4.57 | 36.54 | Spain | Ua-18050 | 32770 | | 1065 | 37460 | AMS |
| Beneito | Aurignacian | -0.47 | 38.70 | Spain | AA-1388 | 33900 | | 1100 | 38660 | AMS |
| Castelcivita | Aurignacian | 15.23 | 40.48 | Italy | Beta-58184 | 32390 | | 490 | 36820 | AMS |
| Covalejos | Aurignacian | -3.93 | 43.39 | Spain | GrA-24220 | 32840 | | 280 | 37170 | AMS |
| Flageolet | Aurignacian | 0.58 | 44.82 | France | GifA-95560 | 32040 | | 850 | 36530 | AMS |
| Fumane | Aurignacian | 10.88 | 45.55 | Italy | Utc-2051 | 32800 | | 400 | 37140 | AMS |
| Geissenklosterle | Aurignacian | 9.78 | 48.40 | Germany | Eth-8268 | 33100 | | 680 | 37570 | AMS |
| Hohle Fels | Aurignacian | 9.73 | 48.37 | Germany | KIA-16036 | 33090 | | 260 | 37350 | AMS |
| Hohlenstein Stadel | Aurignacian | 10.38 | 48.77 | Germany | KIA-13077 | 32270 | | 270 | 36700 | AMS |
| La Quina | Aurignacian | 0.45 | 45.82 | France | Ly-256 | 32650 | | 850 | 37130 | AMS |
| Le Piage | Aurignacian | 0.95 | 45.10 | France | GifA-101260 | 32800 | | 700 | 37220 | AMS |
| Les Renardieres | Aurignacian | 0.37 | 45.83 | France | OxA-12456 | 32170 | | 220 | 36530 | AMS |
| Mitoc Malu Galben | Aurignacian | 27.77 | 46.38 | Romania | GrA-1357 | 32730 | | 220 | 37090 | AMS |
| Mochi | Aurignacian | 7.53 | 43.78 | Italy | OxA-3588 | 32280 | | 580 | 36720 | AMS |
| Oblazowa | Aurignacian | 20.17 | 49.42 | Poland | OxA-4584 | 32400 | | 650 | 36830 | AMS |
| Pena de Candamo | Aurignacian | -6.08 | 43.45 | Spain | no code | 32310 | | 690 | 36750 | AMS |
| Roc de Combe | Aurignacian | 1.35 | 44.75 | France | OxA-1262 | 33400 | | 1100 | 38300 | AMS |
| Sesselfelsgrotte | Aurignacian | 11.79 | 48.94 | Germany | OxA-4245 | 32430 | | 670 | 36860 | AMS |
| Stranska Skala | Aurignacian | 13.68 | 49.82 | Czech Rep. | AA-41479 | 33030 | | 620 | 37420 | AMS |
| Temnata | Aurignacian | 24.05 | 43.17 | Bulgaria | no code | 33000 | | 900 | 37670 | AMS |
| Trou Al Wess | Aurignacian | 5.25 | 50.47 | Belgium | no code | 32325 | | 660 | 36770 | AMS |
| Vogelherd | Aurignacian | 10.07 | 47.95 | Germany | KIA-8970 | 33080 | | 320 | 37350 | AMS |
| Wildscheuer | Aurignacian | 8.17 | 50.40 | Germany | OxA-7390 | 32650 | | 700 | 37070 | AMS |
